# Multi-trophic interactions driving the transmission cycle of *Borrelia afzelii* between *Ixodes ricinus* and rodents: a review

**DOI:** 10.1186/s13071-015-1257-8

**Published:** 2015-12-18

**Authors:** Gilian van Duijvendijk, Hein Sprong, Willem Takken

**Affiliations:** Laboratory of Entomology, Wageningen University and Research Centre, Wageningen, The Netherlands; Laboratory for Zoonosis and Environmental Microbiology, National Institute for Public Health and Environment (RIVM), Bilthoven, The Netherlands; Laboratory of Entomology, Wageningen University and Research Centre, Wageningen, The Netherlands

**Keywords:** *Ixodes ricinus*, *Borrelia burgdorferi*, Trophic interactions, Ecology, Lifecycle, *Apodemus*, *Myodes*, Pathogen transmission

## Abstract

The tick *Ixodes ricinus* is the main vector of the spirochaete *Borrelia burgdorferi* sensu lato, the causal agent of Lyme borreliosis, in the western Palearctic. Rodents are the reservoir host of *B. afzelii*, which can be transmitted to *I. ricinus* larvae during a blood meal. The infected engorged larvae moult into infected nymphs, which can transmit the spirochaetes to rodents and humans. Interestingly, even though only about 1 % of the larvae develop into a borreliae-infected nymph, the enzootic borreliae lifecycle can persist. The development from larva to infected nymph is a key aspect in this lifecycle, influencing the density of infected nymphs and thereby Lyme borreliosis risk. The density of infected nymphs varies temporally and geographically and is influenced by multi-trophic (tick-host-borreliae) interactions. For example, blood feeding success of ticks and spirochaete transmission success differ between rodent species and host-finding success appears to be affected by a *B. afzelii* infection in both the rodent and the tick. In this paper, we review the major interactions between *I. ricinus*, rodents and *B. afzelii* that influence this development, with the aim to elucidate the critical factors that determine the epidemiological risk of Lyme borreliosis. The effects of the tick, rodent and *B. afzelii* on larval host finding, larval blood feeding, spirochaete transmission from rodent to larva and development from larva to nymph are discussed. Nymphal host finding, nymphal blood feeding and spirochaete transmission from nymph to rodent are the final steps to complete the enzootic *B. afzelii* lifecycle and are included in the review. It is concluded that rodent density, rodent infection prevalence, and tick burden are the major factors affecting the development from larva to infected nymph and that these interact with each other. We suggest that the *B. afzelii* lifecycle is dependent on the aggregation of ticks among rodents, which is manipulated by the pathogen itself. Better understanding of the processes involved in the development and aggregation of ticks results in more precise estimates of the density of infected nymphs, and hence predictions of Lyme borreliosis risk.

## Background

*Borrelia burgdorferi* sensu lato (s.l.), a tick-borne pathogen, can cause Lyme borreliosis in humans [1]. *Borrelia burgdorferi* s.l. consists of several genospecies, of which *B. afzelii*, *B. garinii* and *B. burgdorferi* sensu stricto (s.s.) are the main cause of Lyme borreliosis in the western Palearctic [2, 3]. Each of these genospecies is associated with different enzootic lifecycles [4] and clinical manifestations [5]. *Borrelia afzelii* has been mostly associated with skin manifestations, whereas *B. garinii* is considered to be the most neurotropic and *B. burgdorferi* s.s. seems to be the most arthritogenic [6, 7]. Depending on the geographical location, the most common genospecies in *I. ricinus* are *B. afzelii* and *B. garinii* [8–12]. These genospecies are associated with different vertebrate host species. *Borrelia afzelii* is associated with rodents [4, 13–15], whereas *B. garinii* is associated with birds [4, 16]. Because there is sufficient data on the interactions between rodents, ticks and borreliae (in contradiction to the data on birds) and because rodents are the main blood host for larvae [17], this review focusses on *B. afzelii* and rodents.

*Ixodes ricinus* is the principal vector of borreliae in the western Palearctic. This tick has three blood-feeding stages (larva, nymph and adult), which take a single blood meal before moulting to the next stage or laying eggs in the case of an adult female. Adult males do not feed. Larvae can become infected with *B. afzelii* via a blood meal from an infected rodent or via a blood meal from an uninfected host when feeding in close vicinity of a *B. afzelii*-infected tick, a co-feeding infection [18–21]. Rodents can become infected through the bite of an infected tick. It is generally believed that nymphs are responsible for infecting rodents because larvae are rarely infected and adults rarely feed on rodents. Nymphs are also the principle vectors that transmit borreliae to humans [22]. Therefore, the density of infected nymphs affects Lyme borreliosis risk, as was shown in the Nearctic [23]. The density of infected nymphs is determined by the density of nymphs * nymphal infection prevalence.

The interactions between ticks and rodents are complex and can influence pathogen transmission [24, 25]. The development from uninfected larva to infected nymph is a key aspect in the enzootic borreliae lifecycle. Density of larvae is about one order of magnitude higher than the density of nymphs [26, 27]. Nymphal infection prevalence varies temporally and geographically, due to differences in climatic conditions [28], but is about 10 % [9, 12, 29]. As a result, only about 1 % of the *I. ricinus* larvae develops into a borreliae-infected nymph.

**Fig. 1:**
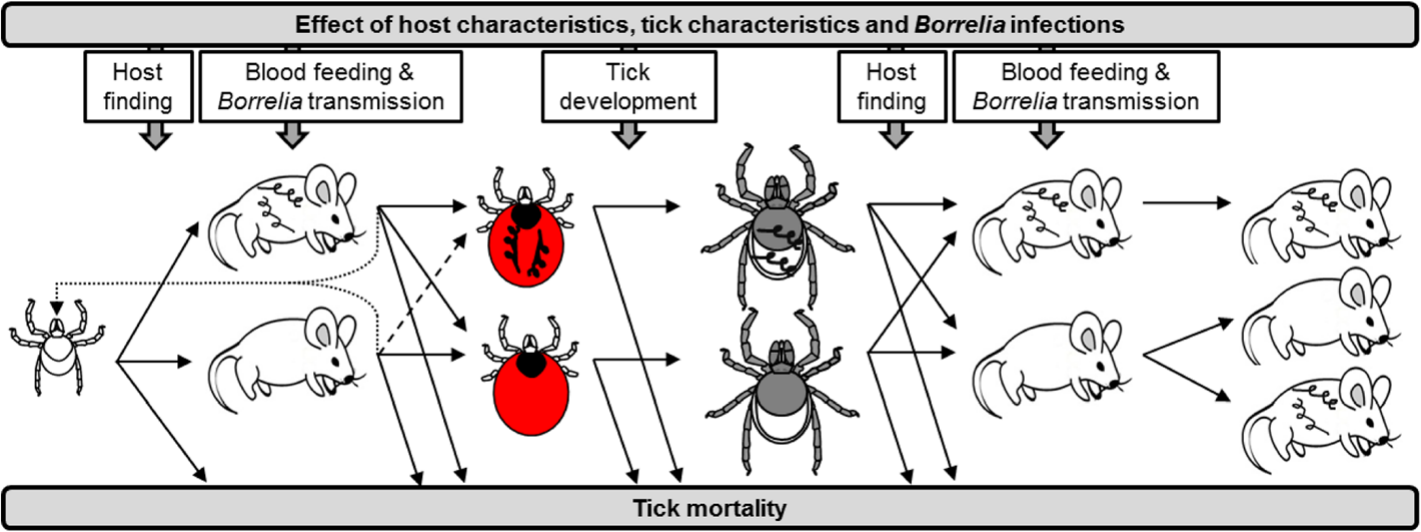
From larva to infected nymph. Schematic overview of the steps involved in the development from an uninfected *Ixodes ricinus* larva to a *B. afzelii*-infected nymph and the transmission process of *B. afzelii* between rodent and tick. Dotted lines indicates continuation of questing after a partial blood meal, dashed line indicates co-feeding transmission

The aim of this review is to give an overview of the major multi-trophic (tick-rodent-*B. afzelii*) interactions that influence the development from an uninfected larva to an infected nymph. This development depends on the success of 1) host attachment of larvae, 2) blood feeding of larvae, 3) borreliae transmission from rodent to larvae, and 4) development from engorged larva to nymph (Fig. 1). Host attachment of nymphs, blood feeding of nymphs and borreliae transmission from nymph to rodent are the final steps to complete the enzootic *B. afzelii* lifecycle and therefore included. The review summarizes the current state of knowledge of the interactions between sub-adult *I. ricinus*, rodents and *B. afzelii* in the western Palearctic and how these interactions affect Lyme borreliosis risk.

### Host attachment

*Ixodes ricinus* feeds on a variety of host species. Each instar of the tick climbs into the vegetation and enters a host-finding stage, termed questing, and senses hosts by CO_2_, host volatiles and vibrations [30–32]. Questing height was lower for larvae compared to nymphs [33]. During questing, water is lost from the tick, which can be reabsorbed when in the litter layer [34]. Attachment to a host is the first major step in the development from larva to nymph, but the chance that a larva encounters a rodent is unknown. Instead, tick burden will be used as a measurement of host-attachment success. Tick burden is determined by tick encounter rate, attachment success, grooming and tick feeding duration [35]. Factors affecting larval and nymphal host attachment are comparable and therefore combined in this paragraph.

#### Host effects

The chance that a questing larva encounters a host affects the density of nymphs and is influenced by the density and activity of hosts [36, 37], which vary between host species. Tick burdens vary between the most common rodent species in Europe; wood mouse (*Apodemus sylvaticus*), yellow-necked mouse (*Apodemus flavicollis*) and bank vole (*Myodes glareolus*). Larval tick burden is higher on wood mice than on bank voles [38–44], which may be caused by differences in ecological niche, activity, home range, grooming activity and immune response [45–47]. Bank voles have an innate and acquired tick resistance resulting in a lower tick attachment success compared to wood mice [45–51]. *Ixodes ricinus* can sense their host by smell [52] and may even be able to distinguish between host species as was shown in the Nearctic for *I. scapularis* [53, 54]. The genetic population structure of *I. ricinus* indicated that the species shows some host specialization [55]. The scale of this specialization is, however, unknown. Tick burden also varies within host species. In general there are many individuals with low tick burdens and few hosts with high tick burdens, feeding the majority of ticks [40, 56], following the 20/80 rule [57, 58]. This intra-species variation can be influenced by sex, age, body mass and activity of the host. In general, tick burden is higher on males compared to females, older rodents compared to younger rodents, heavy weight rodents compared to light weight rodents and active rodents compared to less-active rodents [40, 59–64]. However, these relationships are complex, can be correlated to each other (e.g. males have a greater body weight than females) or interact with each other (tick burdens on females decreased with age whereas they increased on males) [56, 65].

Host preference of *I. ricinus* has not been tested experimentally, but there are examples of other tick species that show an intra-species host preference. *Dermacentor variabilis* preferred the odours from larger and male mice over smaller and female mice [66], *I. arboricola* preferred well developed bird nestlings over less developed nestlings [67], whereas *I. hexagonus* preferred sick hedgehogs over healthy ones [68]. Testosterone can also affect tick burden. It facilitates dominance in wild mice [69] and reduces innate and acquired resistance to ticks [47]. Testosterone levels also differed between rodent species and the level was 10 times higher in wood mice compared to bank voles [47]. High testosterone levels can also reduce tick feeding speed, as was found for ticks feeding on lizards [70].

#### Tick effects

Ectoparasites such as ticks affect the fitness of their hosts in various ways. For example, only 0.17 % blood loss of gerbils (*Gerbillus dasyurus*) resulted in a 16 % increased energy use [71]. This increased energy use should be compensated by an increased energy uptake and therefore host activity, increasing ectoparasite encounter rate. Feeding ticks can consume up to 65 % of the blood from a rodent [40], affecting fitness and activity. Tick feeding can also cause erosion of the ear margin [72], reducing host fitness. Hosts with a larger home range have a higher reproductive success but may also have a higher tick burden, as was shown for *I. scapularis* and *D. variabilis* [64, 73]. Larval tick burdens on rodents increased with increasing densities of questing larvae, but it was suggested that rodents can become saturated with larvae [65]. In addition, the heterogeneity in larval tick burdens on rodents can also be affected by the heterogeneous dispersal of larvae in the environment, increasing the chance of acquiring multiple larvae simultaneously.

#### Borreliae pathogen effects

There is abundant evidence that pathogens can influence their host and/or vector to enhance transmission [74, 75]. Evidence of borreliae manipulating host attachment of ticks is, however, scarce. In the field, borreliae-infected rodents have higher tick burdens compared to borreliae-uninfected rodents [14, 44, 62]. Once infected, spirochaete load did not affect tick burden on rodents [76]. It is unclear whether borreliae can manipulate tick burden (e.g. due to a higher energy demand or altered odour of the rodent) or whether a high tick burden increased exposure risk to borreliae*.* Hosts with high nymphal tick burdens have a higher chance of becoming infected with borreliae and rodents infested with nymphs have higher larval tick burdens than rodents without nymphs [40, 60, 77]. A borreliae infection does not affect rodent survival [78, 79], but a specific borreliae antibody response altered foraging behaviour of white footed mice in the Nearctic [80, 81], which may increase tick encounter rate. There is also evidence that borreliae can influence host-tick contact rate when in the tick. A borreliae infection in adult female *I. ricinus* increased host finding efficacy [82]. In addition, borreliae-infected nymphs had higher energy reserves and spent more time questing for a host compared to borreliae-uninfected nymphs [33, 83–87]. These effects were influenced by *B. burgdorferi* s.l. genospecies [86]. However, all these results are from field-collected ticks and observed differences may, therefore, have been caused by characteristics of the host on which the ticks fed as larvae (e.g. species, tick burden, immune response). If, for example, tick burden positively affects blood meal size (see below), borreliae*-*infected ticks will have a higher fat content due to the higher tick burden of infected rodents, while this was not caused by the borreliae infection of the tick.

### Blood feeding

Once a sub-adult tick has encountered a rodent, it needs to find a feeding site, bite the host and acquire a blood meal. It is generally assumed that each larva takes only one bloodmeal before moulting to a nymph. Factors affecting larval and nymphal blood feeding are comparable and therefore combined in this paragraph.

#### Host effects

Blood feeding of *I. ricinus* is a complex process with major events occurring within the tick [45] and can be influenced by host species. Blood meal size and percentage of fully engorged ticks are larger for larvae feeding on *Apodemus* mice, which therefore moult into larger nymphs, compared to larvae that fed on bank voles [40, 41, 47, 88]. Exposure to tick saliva caused acquired tick resistance in bank voles, resulting in a decreased blood ingestion speed [47–50], whereas this was increased in yellow necked mice [48]. In addition, feeding duration had a positive effect on blood meal size in bank voles, but not in wood mice [41]. The effect of acquired tick resistance on tick feeding in *I. ricinus* was also found for rabbits [89].

#### Tick effects

Tick saliva has anti-haemostatic, anti-inflammatory, and immunosuppressive effects on the host’s immune system, facilitating blood consumption of ticks [51, 90]. A larger tick burden results in more tick saliva and a higher immunosuppressive effect, which could therefore facilitate blood feeding of ticks. As a result, a high tick burden increased feeding success of *I. ricinus* feeding on wood mice and bank voles [47].

#### Borreliae pathogen effects

There are indications that aborreliae-infection results in an increased blood meal size of larvae. Infected engorged larvae collected from wood mice were heavier and moulted into larger nymphs compared to uninfected larvae [85]. The higher energy reserve of borreliae-infected nymphs (see above) is also likely to be a result of a larger blood meal during the larval stage. However, these differences could have been caused by a higher tick burden of infected hosts, affecting the immune response of the host (see above). In an artificial feeding system, blood meal size of nymphs decreased when fed *Bartonella*-infected blood compared to *Bartonella*-uninfected blood, whereas feeding duration was not affected [91].

### Development from engorged larva to nymph

A larva that acquired a complete blood meal detaches from the host to digest its blood meal and moult into a nymph. When the larva acquired borreliae during this blood meal, it will emerge as an infected nymph after moulting.

#### Host effects

Moulting success from larva to nymph can be influenced by host species and was higher for larvae that naturally attached to field collected *Apodemus* mice compared to bank voles [13, 92], but the opposite happened for laboratory reared ticks [92]. After multiple infestations, moulting success remained stable in *Apodemus* mice but declined in bank voles; this effect was abolished, however, when testosterone levels were increased [47, 48]. A reduced moulting success was also found for *I. trianguliceps* feeding on bank voles [49] and may have been caused by a difference in blood meal success (see above) because partially-engorged larvae failed to moult [48].

#### Tick effects

Endosymbionts are widespread among arthropods [93, 94]. The effects of endosymbiots have not been investigated in *I. ricinus*. However, in the Nearctic they have been shown to influence tick fitness [95] and the colonization of borreliae in the tick [96]. The relationship between the tick microbiome and tick survival and borreliae transmission are far from understood [97] and has not been investigated in *I. ricinus*.

#### Borreliae pathogen effects

During moulting, borreliae spirochaetes survive in the midgut lumen of the tick and persistence until the next feeding is crucial for successful transmission [98, 99]. The interactions between the tick’s defence mechanisms and borreliae during moulting have been reviewed [100]. It was shown that in the case of *I. scapularis*, even though borreliae load is reduced five fold during moulting and remained stable at <300 spirochaetes in the emerged nymph [101], spirochaete genetic population structure was not affected during moulting [102]. Whether a borreliae infection affects interstadial development from *I. ricinus* larva to nymph is unknown.

### *Borrelia* transmission from rodent to larva

To maintain the enzootic borreliae lifecycle*,* rodents must feed both larvae and nymphs and an infection acquired by a larva must be transstadially transmitted during the moult to a nymph. Feeding larvae can also become borreliae-infected through co-feeding with an infected nymph on a host without a systemic infection [18–20]. However, because rodents are the main host used by larvae and can be systemically infected with borreliae*,* the effect of co-feeding transmission on the zoonotic life cycle of borreliae appears to be limited. The chance that a larva acquires borreliae from a host is determined by the borreliae prevalence in the host community, which is influenced by the probability that infected nymphs feed on the host, host susceptibility to the pathogen and the ability of the host to maintain the infection. The survival of borreliae within the host and tick, and transmission between them, are underpinned by molecular mechanisms, which have been reviewed [35, 103].

#### Host effects

Not all host species used by *I. ricinus* are borreliae reservoirs and there is high variation in transmission efficiency among reservoir hosts. Rodents are associated with *B. afzelii* [4, 13–15]. *Borrelia burgdorferi sensu stricto*, *B. bavariensis* and *B. spielmanii* are also associated with rodents, but have a lower infection prevalence in questing nymphs [9, 104, 105]. Rodents can also be co-infected with multiple *B. burgdorferi* s.l. genospecies [15, 106, 107]. However, these different genospecies were not necessarily acquired through the bite of one nymph co-infected with multiple genospecies, but could be transmitted by multiple infected nymphs. Large mammals like roe deer and red deer are hosts for ticks, but incompetent for borreliae transmission, presumably because of anti-borreliae immune responses [108, 109]. In the Nearctic, rodent infection rate (percentage of infected hosts) and host infectivity (percentage of uninfected larvae that acquire a borreliae infection during feeding on an infected host) are positively correlated and vary between host species [110]. Whether this is also true for the western Palearctic is not known. Rodent infection rate is lower in wood mice compared to bank voles and varies temporally and geographically [13, 15, 44, 111]. Rodent infection rate can also differ between sexes and was higher in males compared to females [112], which was likely due to higher nymphal tick burdens on males, increasing exposure to borreliae. Infected rodents stay infective throughout their life resulting in a higher rodent infection rate of older rodents compared to younger rodents [113]. Borreliae infection prevalence of ticks fed on wild rodents was lower in April (1.2-10.5 %) compared to June/July (15.1–17.5 %) and did not increase until October [39], which is probably explained by a lower rodent infection rate caused by a lower exposure to borreliae-infected nymphs during winter compared to spring, summer and autumn.

Host infectivity is also influenced by host species and is lower in mice compared to voles [13, 39, 114, 115]. The differences between wood mice and bank voles can be caused by the number of borreliae-specific antibodies in the host, which correlated negatively to infectivity [116]. Even though infections were not lost, host infectivity can vary over time and decreases since inital infection of the rodents [112, 117]. A correlation between host body size versus infectivity and spirochaete burden in feeding ticks has not been tested for *I. ricinus*. However, this correlation was negative at host species level for *I. scapularis* [118]. These authors suggested that this was caused by a difference in time between inoculation and putative threshold for infectiousness.

#### Tick effects

The aggregation of ticks among hosts results in an increased borreliae transmission when larval and nymphal tick burdens are correlated. In addition, infectivity increased with successive larval infestations and larval tick burden [39, 113], increasing the contribution of these heavily infested individuals. Host infectivity of host associated *B. burgdorferi* s.l.-genospecies (*B. garinii* and *B. valaisiana* for birds) increased with successive infestations with field collected *I. ricinus* nymphs, whereas infectivity of genospecies associated with other hosts (*B. afzelii* for birds) decreased, suggesting a possible developed resistance [119]. The effect of tick burden on infectivity may be caused by the immunosuppressive effect of tick saliva on the rodent immune system [51, 90], resulting in an increased infectivity. However, infectivity of bank voles was reduced at sites with high tick densities [39]. Borreliae transmission from host to ticks increases with feeding time and started 2–8 h after tick attachment [120]. *Borrelia afzelii* has to survive the tick immune system during blood digestion, moulting and migration via the haemolymph to the salivary glands [121]. Nymphal infection prevalence had a positive effect on infection prevalence of larvae fed on rodents [39], most likely due to a higher exposure of rodents to infected nymphs.

#### Borreliae pathogen effects

Rodent infection rate varies between *B. burgdorferi* s.l. genospecies and is highest for *B. afzelii*, followed by *B. burgdorferi* s.s. and *B. garinii* [15, 44]. Hosts can transmit multiple genospecies to feeding ticks [119], but host infection does not necessarily mean that the spirochaetes are transmitted to feeding ticks, as was shown for rodents infected with *B. garinii* in internal organs, which only transmitted *B. burgdorferi s.s.* to feeding larvae [4]. Spirochaete load of *B. burgdorferi s.s*. was higher when mice were co-infected with *B. garinii*, compared to an infection with only *B. burgdorferi s.s*., whereas the opposite happened for *B. garinii* [122], indicating interactions between the two genospecies while in the same host, which benefits *B. burgdorferi s.s*. Time until infectiousness also differs between genospecies; wood mice became infectious with *B. afzelii* in fewer days post infection and with a higher infectivity compared to *B. burgdorferi s.s*. [79, 117]. *Borrelia burgdorferi s.s*. was found only in rodents during tick activity, but not during winter [13], suggesting that these reservoir hosts are not a permanent reservoir for all genospecies and can lose infections, as was shown in the Nearctic for *Peromyscus leucopus* [123]. However, *B. burgdorferi s.s*. was also only found in mouse blood up to eight days after inoculation, whereas spirochaetemia lasted up to six weeks after inoculation [122]. Infectivity also differs between borreliae isolates, as was shown for *B. afzelii* [124]. The increased host’s infectivity with time (see above) was also genospecies dependent and increased faster in *B. afzelii* compared to *B. burgdorferi s.s*. [120]. *Borrelia afzelii* (rodent associated) on the one hand and *B. garinii* and *B. valaisiana* (bird associated) on the other hand infect adult *I. ricinus* on a mutualistic exclusive way; they co-occurred less frequently than expected compared to co-infections with *B. garinii* and *B. valaisiana* [125]. Whether these different genospecies were transmitted during a single feed on one host or two feeds on separate hosts (as larva and nymph) is unclear, but it seems likely that *B. afzelii* in nymphs feeding on birds was negatively selected by host complement in the midgut of feeding ticks [126]. Strong genetic differentiation was observed between *B. burgdorferi* s.l. genotypes infecting different rodent species, suggesting host specificity of borreliae populations [127]. Spirochaete load at the feeding site positively influenced host infectivity [76, 102] and rodents with a high infectivity transmit more borreliae spirochaetes to larvae compared to the larvae fed on rodents with a lower infectivity [113]. However, even though spirochaete load was ten times higher in voles compared to mice, this did not result in a higher infectivity of voles compared to mice and this was probably due to a larger blood meal size on mice [76]. If a high spirochaete load in rodents results in a high spirochaete load in feeding ticks, infectivity from tick to host may also be enhanced. Spirochaete load in rodents and feeding ticks were, however, not correlated [76].

### *Borrelia* transmission from nymph to rodent

Rodents acquire a borreliae infection through the bite of an infected tick and not via vertical transmission from female to offspring, as was shown for the Nearctic reservoir host *Peromyscus leucopus* [128, 129]. *Ixodes ricinus* larvae are rarely infected with *B. burgdorferi* s.l. [120] and adults rarely feed on rodents [44], suggesting that nymphs are responsible for transmitting borreliae to rodents. After a borreliae-infected nymph attaches to a host, the borreliae spirochaetes in the midgut multiply and migrate through the midgut wall via the haemolymph to the salivary glands, from which they may be inoculated with the tick saliva into the host [130, 131]. Borreliae transmission from nymph to host is positively correlated with feeding duration of the tick and in general does not occur before 24 h of feeding [132, 133]. However, borreliae can be transmitted as early as after 16–17 h of feeding [134], which may have been caused by a systemic borreliae infection in the tick [135]. Once spirochaetes have been inoculated into the host’s skin, they remain at the inoculation site and disseminate after a few days, as was shown for *B. burgdorferi* s.s. in the Nearctic [136]. Borreliae have been detected in skin, blood, joints, spleen, heart, liver, urinary bladder, kidney and nervous system of vertebrate hosts [137–140].

#### Host effects

Not all host species are susceptible to each *B. burgdorferi* s.l. genospecies, due to differences in complement-mediated sensitivity of the spirochaetes to host serum [126]. *Borrelia afzelii* is mainly associated with rodents [4, 14], *B. garinii* and *B. valaisiana* with birds [4, 16], *B. lusitaniae* with lizards [141] and *B. spielmanii* with dormice [142]. As a result, an infected tick that feeds on a host that is incompetent for the concerning genospecies appears to lose its infection [109, 143, 144]. However, this is not always the case, as was shown for *B. afzelii* in songbirds [119]. In the Nearctic, pre-exposure of rodents to *I. scapularis* reduced susceptibility to borreliae, irrespective of an acquired tick immunity [145, 146]. This suggests that nymphal infection prevalence can influence rodent infection rate directly (a low nymphal infection prevalence reduces borreliae exposure to the host) or indirectly (a low nymphal infection prevalence reduces host susceptibility to borreliae) and that a high larval tick burden may reduce rodent infection rate by acquired immunity. This has not been investigated for *I. ricinus*, but indeed, rodent infection rate of white footed mice was ten times higher in periods with high risk of exposure to *I. scapularis* nymphs compared to a period of low risk [147]. Susceptibility to borreliae differed between bank vole individuals and was influenced by their genetic variation [112].

#### Tick effects

When borreliae-infected *I. ricinus* nymphs can feed to repletion, transmission success from nymph to host was almost 100 % [134]. Nymphs do not need to have acquired the borreliae spirochaete(s) during a blood meal in the larval stage. It was shown for *I. scapularis* that nymphs can acquire borreliae during an interrupted feeding of 16 h and can infect another host after 3–5 days without first moulting to the next stage [148]. However, larvae that fed partially (18 h) on a borreliae-infected host were not infectious during a second blood meal five weeks after the initial feeding, whereas borreliae were transmitted after they moulted into nymphs [149]. Partially-fed ticks can arise by tick immunity of the host [48]. In addition, grooming of the host or host mortality may also result in partially-fed ticks. However, whether this can also happen in *I. ricinus* and frequencies of naturally occurring partially-fed larvae and nymphs are unknown.

#### Borreliae pathogen effects

Ticks can be co-infected with more than one *B. burgdorferi* s.l. genospecies [8, 9, 12, 135, 150], with up to 45 % of infected ticks harbouring multiple genospecies [151]. Even though adult ticks have taken an additional blood meal, co-infection prevalence was not higher in adults compared to nymphs [12], which may be caused by the clearance of the genospecies acquired during the first blood meal by the ingestion of host complement during the second blood meal. Therefore, at least in the case of the nymphal stage, co-infections are likely to be acquired during one single blood meal from a co-infected host. The majority of co-infected nymphs is therefore co-infected with two genospecies that can co-occur in the same host [151, 152]. Spirochaete load in nymphs co-infected with genospecies that share vertebrate hosts was equal to or higher than the additive expectation, whereas this was lower for genospecies associated with different reservoir hosts [152]. Spirochaete load in infected ticks was higher for *B. garinii* and *B. bavariensis* compared to *B. afzelii* [83, 152]. Even though all spirochaete clones present in the host were transmitted to the feeding larvae and survived moulting to the nymphal stage, only a small fraction of the spirochaetes in the tick’s midgut are transmitted from nymph to host during feeding [102, 153]. Whether a high spirochaete load in infected nymphs results in a greater transmission success to a host during feeding is unknown. When injected intradermally, only 10 cultured borreliae spirochaetes were enough to infect a mouse [154].

## Conclusions

Understanding the factors that affect the density of infected nymphs increases our knowledge on Lyme borreliosis risk. The development from questing *I. ricinus* larva to borreliae-infected nymph is affected by many biological and ecological factors. The existence of different *B. burgdorferi* s.l. genospecies and heterogeneity between and within genospecies makes the tick-rodent-borreliae interactions complex. The development from larva to nymph, regardless of a borreliae infection, affects nymphal density and appears to be successful in only 10 % of the time [155]. The chance that a larva encounters a rodent affects the density of nymphs and is influenced by rodent density, which differs between rodent species and varies spatially and temporally [39, 63]. Even though it is a major step in the development, there is no data of the chance that a larva actually encounters a rodent or any other host. Host encounter rate may not be fully dependent on external factors but may be affected by the tick too, e.g. when larvae are attracted to a rodent trail. Nymphs for example, are attracted to perches that have been scented with rodent odour [52]. It was shown that ticks prefer odours from certain hosts over others. However, it is unknown if questing larvae can afford to reject a non-preferred host, risking the possibility of not acquiring any blood meal and starving to death.

Rodents with high larval tick burdens, which are major contributors to the density of nymphs, have in general also higher nymphal tick burdens, making them more likely to be infected with borreliae. As a result, these rodents are even larger contributors to the density of infected nymphs. We hypothesize that this aggregation increases nymphal infection prevalence and that this aggregation is therefore necessary for the maintenance of the enzootic borreliae lifecycle. There is some evidence that the aggregation of ticks can be caused by borreliae [14, 44]. Therefore, the chance that a larva acquires a blood meal from an infected rodent may not solely be the effect of the density of infected rodents, e.g. when borreliae-infected rodents are more active than uninfected rodents, or when questing larvae prefer—the odours from—borreliae-infected rodents, the chance of acquiring a borreliae infection is greater than the effect of rodent density alone. Even though the only experimental study conducted on this subject showed no effect of a borreliae infection in rodents on tick attraction [52], there are many examples of parasites manipulating their hosts [74, 75]. Therefore, understanding rodent or tick manipulation by borreliae requires more experiments with experimentally infected rodents and ticks to exclude biases from differences in rodent characteristics on physiological or behavioural differences between infected and uninfected rodents and ticks.

The borreliae lifecycle does not only benefit from aggregation of larvae on (borreliae-infected) rodents, but also from the successful development from larvae to nymph and the chance that aborreliae-infected nymph encounters a (borreliae-uninfected) rodent. Whether borreliae can affect interstadial tick development (e.g. moulting success) is unknown and requires more research to overcome a bias in the effect of the 90 % mortality during development from larva to nymph [155].

Tick survival, rodent density, rodent infection rate and host infectivity are major factors affecting the borreliae lifecycle, whereas only the first two directly affect the tick lifecycle. These factors also interact with each other, e.g. even though infection rate and infectivity of bank voles was higher, the higher tick burden on wood mice and moulting success of ticks fed on wood mice made wood mice more important contributors to the density of infected nymphs [13]. The tick lifecycle clearly benefits from a high density of nymphs, whereas the borreliae lifecycle benefits from a high nymphal infection prevalence. However, the borreliae lifecycle also benefits from a high density of nymphs, when this will lead to a higher density of larvae and therefore a higher chance of borreliae transmission from rodent to tick and vice versa. There is evidence that borreliae can affect tick survival, increasing the density of nymphs and therefore enhancing its own lifecycle. Therefore, in addition, the tick lifecycle also benefits from a high nymphal infection prevalence.

More knowledge on these multi-trophic interactions helps to obtain better estimates of the Lyme borreliosis risk. This review showed the various factors that contribute to the density of infected nymphs, and how they interact. These results, together with the effect of abiotic factors, could be mathematically modelled to determine the key processes that determine the density of infected nymphs, and thereby Lyme borreliosis risk.
